# The Impact of the COVID-19 Pandemic on Avoidance of Health Care, Symptom Severity, and Mental Well-Being in Patients With Coronary Artery Disease

**DOI:** 10.3389/fmed.2021.760265

**Published:** 2021-12-15

**Authors:** Nathalie Maehl, Markus Bleckwenn, Steffi G. Riedel-Heller, Sebastian Mehlhorn, Stefan Lippmann, Tobias Deutsch, Anne Schrimpf

**Affiliations:** ^1^Department of General Practice, Faculty of Medicine, Leipzig University, Leipzig, Germany; ^2^Institute of Social Medicine, Occupational Health and Public Health (ISAP), Leipzig University, Leipzig, Germany; ^3^Private Practice, Delitzsch, Germany

**Keywords:** coronary heart disease (CHD), SARS-CoV-2, Disease Management Program (DMP), primary care, treatment-seeking, anxiety, depressive symptoms, angina pectoris

## Abstract

The COVID-19 pandemic affected regular health care for patients with chronic diseases. However, the impact of the pandemic on primary care for patients with coronary artery disease (CAD) who are enrolled in a structured disease management program (DMP) in Germany is not clear. We investigated whether the pandemic affected primary care and health outcomes of DMP-CAD patients (*n* = 750) by using a questionnaire assessing patients' utilization of medical care, CAD symptoms, as well as health behavior and mental health since March 2020. We found that out of concern about getting infected with COVID-19, 9.1% of the patients did not consult a medical practitioner despite having CAD symptoms. Perceived own influence on infection risk was lower and anxiety was higher in these patients compared to symptomatic CAD patients who consulted a physician. Among the patients who reported chest pain lasting longer than 30 min, one third did not consult a medical practitioner subsequently. These patients were generally more worried about COVID-19. Patients with at least one worsening CAD symptom (chest pain, dyspnea, perspiration, or nausea without apparent reason) since the pandemic showed more depressive symptoms, higher anxiety scores, and were less likely to consult a doctor despite having CAD symptoms out of fear of infection. Our results provide evidence that the majority of patients received sufficient medical care during the COVID-19 pandemic in Germany. However, one in ten patients could be considered particularly at risk for medical undersupply and adverse health outcomes. The perceived infection risk with COVID-19 might have facilitated the decision not to consult a medical doctor.

## Introduction

The coronavirus-19 disease (COVID-19) has been causing a global pandemic and developed into a burden for health care systems worldwide (1). The pandemic not only affected patients who suffered from the new disease, but potentially left all patients needing regular treatment or preventive consultations in any medical sectors undersupplied due to insufficient capacities of the health care system. To deal with COVID-19 patients and to reduce infection risks, many planned procedures and preventive consultations concerning all medical services had to be canceled or postponed, affecting in particular patients with chronic diseases (2–4). To date, information on the extent of suspended or postponed primary care consultations of patients with chronic diseases—for instance with chronic heart diseases—is sparse.

Patients with chronic coronary artery disease (CAD) are especially vulnerable to an undersupply of medical care as the pathophysiological process of CAD leads to a higher risk for acute myocardial infarctions (AMI) (5). Still, little research is available on the impact of the pandemic on primary care in chronic CAD patients. Clinical data showed that hospital admissions for AMI decreased since the outbreak of the COVID-19 pandemic in Germany (3, 6, 7). However, an increase in severe AMI cases with higher troponin levels, mortality, major cardiac complications, and a critical time delay from onset of symptoms to first medical contact has been observed compared to pre-pandemic times (6, 8, 9). Importantly, over 50% of these AMI patients reported a pre-existing CAD. A comparable development of AMI cases has also been reported in other countries (10–16).

The underlying causes of reduced or potentially delayed AMI admissions since the outbreak of the COVID-19 pandemic are still under debate (17). It has been discussed that symptomatic patients might have suspended or delayed a hospital visit during the pandemic out of fear of infection with the new respiratory disease. Indeed, AMI patients who delayed first medical contact after symptom onset reported COVID-19-related reasons for delayed presentation (12, 18).

In the outpatient sector, no data are available on the impact of the perceived infection risk on CAD patients' decision to consult a medical doctor. In Germany, currently 1.9 million of these patients are enrolled in a structured treatment and prevention program called Disease Management Program (DMP) for CAD. The DMP includes regular quarterly or biannually consultations with the patient's general practitioner (GP) to adjust medication, discuss disease related lifestyle issues to reduce risk factors, and monitor the progression of the CAD (19). CAD patients enrolled in the DMP have a significantly lower risk of death, better adjustment of and adherence to medication, and received more counseling on health behavior (19–21). However, to reduce the risk of infection with COVID-19 in high-risk populations, the Federal Joint Committee decided in March 2020 that interventions in the DMPs could have been suspended or canceled (22). The impact of this decision, either on protecting risk groups from a COVID-19 infection or on the maintenance of sufficient treatment of patients with chronic diseases, is still unknown (3).

To our knowledge, no study on DMP-CAD patients' treatment-seeking behavior during the COVID-19 pandemic is available so far. Therefore, we invited patients enrolled in the DMP-CAD to participate in our explorative questionnaire-based study to investigate if and to what extent the COVID-19 pandemic affected primary care for these patients. We assessed whether DMP-CAD patients made use of primary care during the pandemic compared to the pre-pandemic period, especially in case of prolonged or severe symptoms of their chronic heart disease. Additionally, potential reasons for delayed or canceled consultations were assessed. As aggravated CAD symptoms lead to a higher risk for cardiac events (23), we examined patients' CAD-related symptoms and whether they worsened since the outbreak of the pandemic. Further, behavioral factors—such as smoking, heavy alcohol consumption, and physical inactivity—have a significant effect on adverse health outcomes and mortality in CAD patients (24, 25). We therefore examined changes in health behavior since the outbreak of the pandemic. In addition, CAD patients' compliance with hygiene measures, as well as risk perception of COVID-19, were investigated. Finally, information on depressive symptoms and anxiety was collected as they have been independently associated with the severity of functional impairment, adverse health behavior, lower adherence to therapy, and lower quality of life in patients with CAD (26–30).

## Methods

### Recruitment and Procedures

**Figure 1:**
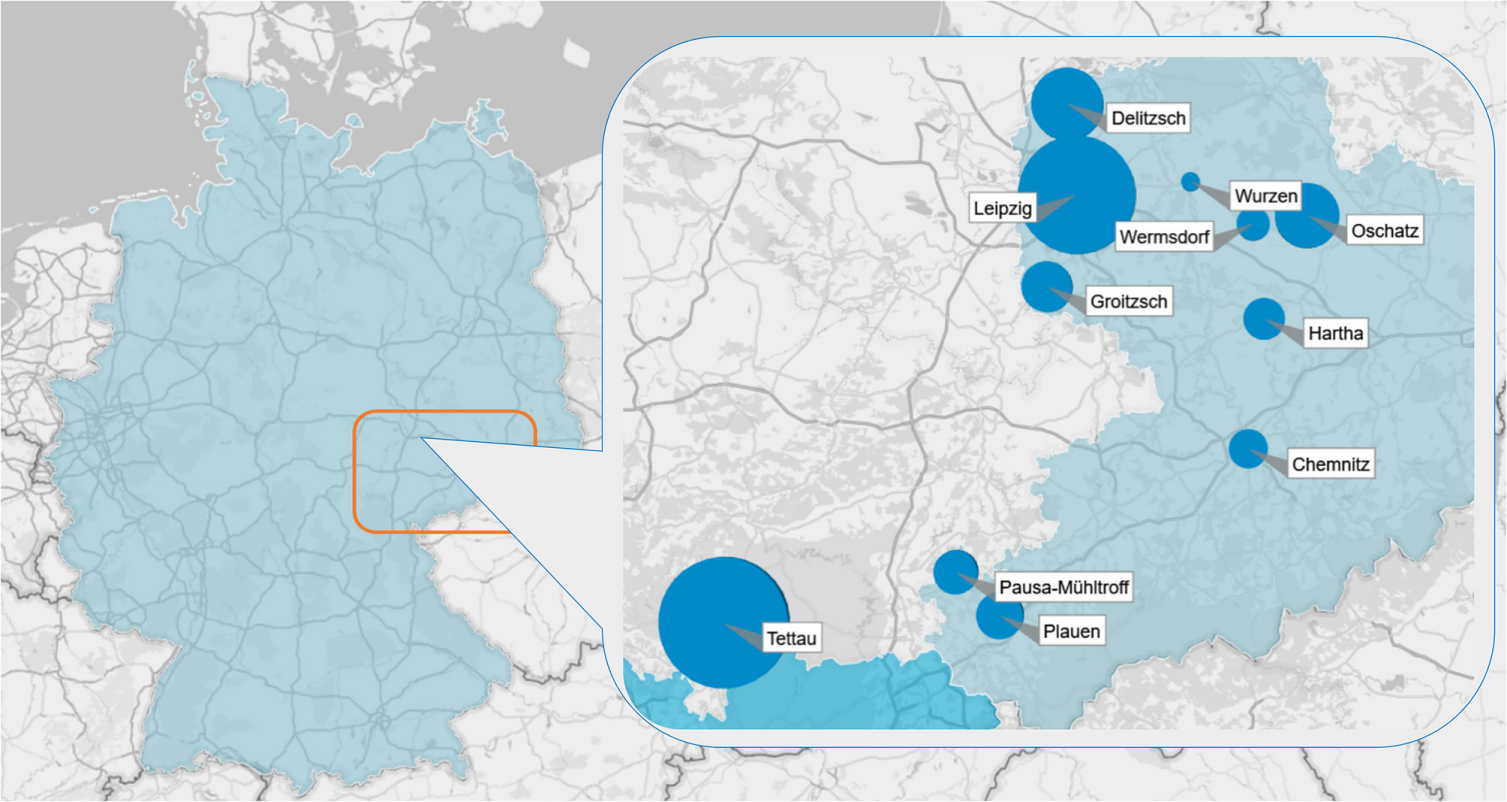
Data were collected in Central Germany. The size of the blue dots indicates the share of patients with coronary artery disease in each location.

**Figure 2:**
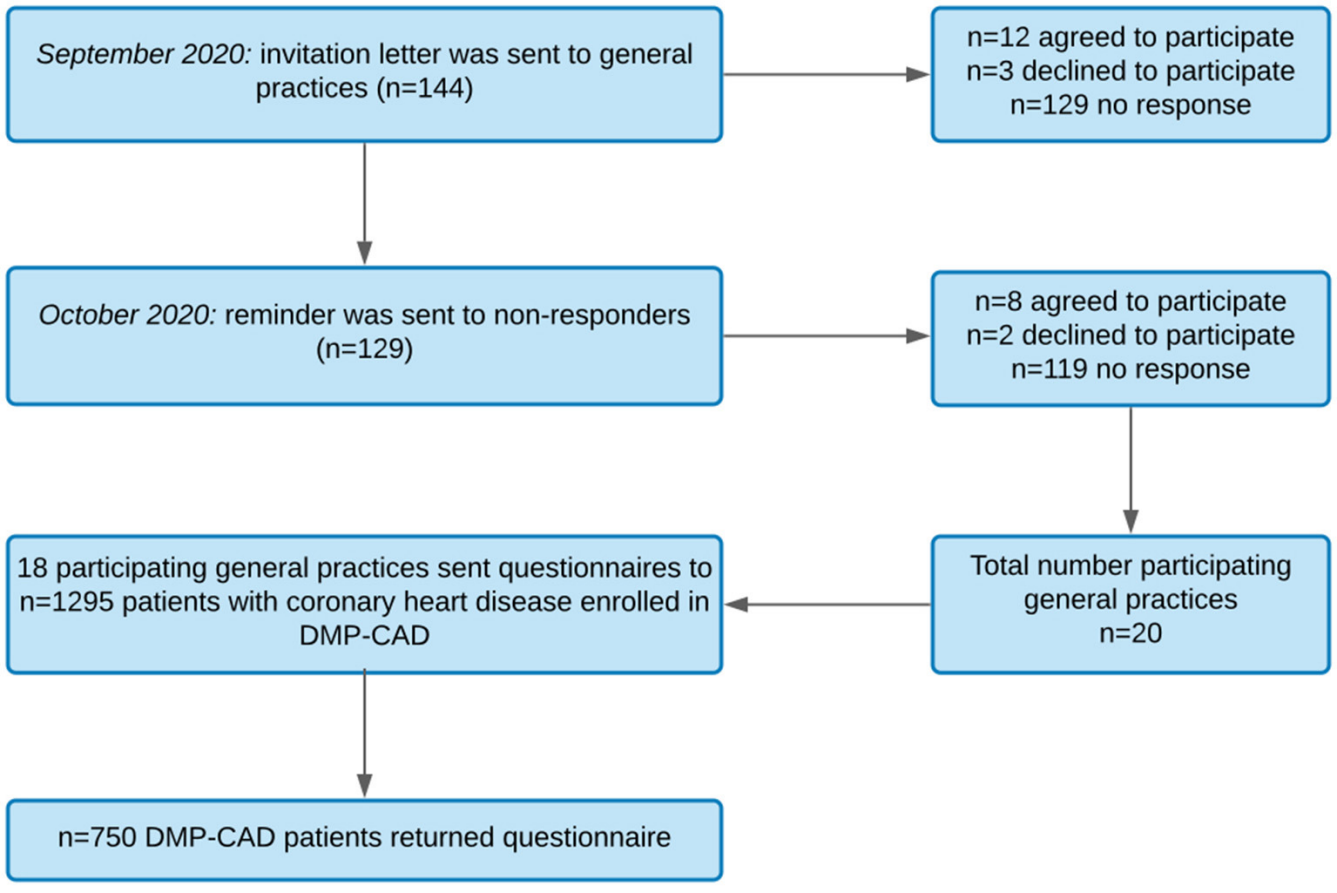
Flowchart of recruitment process. DMP-CAD, Disease management program for coronary artery disease.

The data were collected in Central Germany (Figure 1) between October 2020 and February 2021. To recruit patients enrolled in the DMP-CAD, 144 GP practices were invited by letter to voluntarily participate in this study and to send a questionnaire to their DMP-CAD patients. In September 2020, the first invitation letter including study information, a short questionnaire, and an informed consent form was sent to GP practices. In October 2020, a reminder containing the same documents was sent to non-responders of the first wave according to the recommendations of Edwards et al. (31) (see recruitment process in Figure 2).

Participating GPs were asked to sign the informed consent form, complete a GP questionnaire, and return these documents via mail or fax. The GP questionnaire comprised three questions: (1) total number of patients enrolled in the DMP-CAD, (2) number of DMP-CAD patients with cognitive impairments, (3) numbers of DMP-CAD visits in their practice in the quarters 1 to 4 in 2019 and quarters 1 to 2 in 2020.

In a second step, participating GPs received for each eligible DMP-CAD patient (total number of DMP-CAD patients minus the number of DMP-CAD patients with cognitive impairments) a stamped envelope containing study information, instructions, a stamped return envelope, and a questionnaire. GPs were asked to address the envelopes and send them to their patients. To ensure anonymity, personally identifiable information from patients remained in the practice and was inaccessible to the researchers. In addition, GPs also received a second GP questionnaire assessing: (1) number of DMP-CAD patients who got a study letter, (2) sex of contacted patients, (3) alternative consultation options offered during the pandemic, (4) perceived decrease in DMP examinations during the pandemic.

### GP Sample

Of 144 invited GPs, 20 GPs agreed on study participation. Additional two GPs dropped out after receiving the patient questionnaires. Ultimately, 18 GPs sent study information to their DMP-CAD patients and returned the first and second GP questionnaire. Between September and December 2020, GPs sent the envelopes to their eligible patients. Percentages, means, and standard deviations for GP sample characteristics can be found in Table 1.

**Table 1:** Sample characteristics and questionnaire results of participating general practices.

**n**	18
**Sex**	50%♀
**Mean number of enrolled CAD patients**	89.4 ± 98.7 (12–424)
**Mean number of CAD patients' DMP consultations per quarter**	
**Quarter 1 to 4 in 2019**	82.1 ± 89.9 (6–394)
**Quarter 1 to 2 in 2020**	87.1 ± 98.1 (7–421)
**Perceived decrease in CAD-DMP consultations since March 2020**	31.3%
**Alternative consultation options offered to patients**	
**Separate office hours**	47.1%
**Telephone consultation**	70.6%
**Video consultation**	17.6%
**none**	23.5%

*Data are presented as mean, standard deviations, range, and percentage (n/n_valid_)*.

### Patient Sample

Inclusion criteria for patient recruitment were being enrolled in the DMP-CAD and being fluent in the German language. Patients with a recent history of cognitive impairments were not contacted. In total, 1,295 DMP-CAD patients were recruited and received an envelope. After completing the questionnaire, patients were asked to put the questionnaire in the stamped return envelope and send it back to the Department of General Practice (DGP), Leipzig University. Between October 2020 and February 2021, 751 patients returned the questionnaire (58.0% total response rate). One patient stated that he or she did not have a heart disease and was excluded, so 750 patients were included in the analyses. Mean age was 73.3 years with 32.8% of the participants being female. Percentages, means, and standard deviations for sample characteristics can be found in Table 2. The characteristics of our CAD patients are similar to the DMP-CAD patient sample in a recent German study (20). In their study, CAD patients were mostly male (66.9%), often had co-morbidities such as diabetes mellitus type 2 (41.7%), and showed a similar age distribution with a mean age of 73.7 years.

**Table 2:** Sociodemographic and medical characteristics of the patient sample.

	**Total**	**Age group 40–59 years**	**Age group 60–79 years**	**Age group 80–96 years**	**Age group comparison**	
					***p***	**χ2**
n	750	83	400	242	
Age	73.3 ± 10.3	54.63 ± 3.9	70.6 ± 5.5	84.1 ± 3.4	
Sex	32.8%♀	22.4%♀	28.0%♀	44.1%♀	**<0.001**	20.49
Occupation					**<0.001**	309.72
Working	14.2%	78.3%	9.0%	0.4%		
Unemployed	2.6%	13.3%	2.0%	0.0%		
Retired	83.3%	8.4%	89.0%	99.6%		
Education					**0.005**	14.48
Primary	3.5%	1.2%	3.1%	4.3%		
Secondary	78.1%	89.2%	80.6%	70.7%		
Tertiary	18.4%	9.6%	16.4%	25.0%		
Area					**<0.001**	24.00
City	23.8%	16.5%	19.5%	32.2%		
Small town	26.8%	17.7%	28.7%	28.3%		
Rural	49.4%	65.8%	51.8%	39.6%		
Living alone	22.0%	18.1%	17.0%	31.4%	**<0.001**	19.09
Care level	16.5%	6.5%	9.8%	30.3%	**<0.001**	47.14
CAD symptoms (CCSA)					0.158	9.30
Grade I	58.6%	57.7%	61.1%	54.3%		
Grade II	12.6%	11.5%	12.8%	12.6%		
Grade III	17.1%	15.4%	13.9%	22.9%		
Grade IV	11.7%	15.4%	12.3%	10.3%		
CAD risk stratification					0.330	2.22
Low risk	92.4%	88.5%	93.1%	93.3%		
High risk	7.6%	11.5%	6.9%	6.7%		
COVID-19 infection	0.8%	1.2%	1.3%	0.0%	0.163	3.32
Other chronic diseases						
Asthma	6.1%	4.8%	7.0%	5.4%	0.714	0.81
Breast cancer	3.3%	1.2%	3.0%	4.5%	0.387	2.08
COPD	7.7%	2.4%	9.5%	6.2%	0.052	5.91
Type 1 DM	2.1%	3.6%	2.3%	1.7%	0.536	1.34
Type 2 DM	27.4%	9.6%	29.3%	29.0%	**0.001**	14.25

*Data are presented as mean, standard deviations, and percentage (n/n_valid_). Age was not available for 25 patients. CAD, coronary artery disease; CCSA, Canadian Cardiovascular Society Angina Classification; COPD, Chronic obstructive pulmonary disease; DM, Diabetes mellitus. The bold p-values highlight a significant result*.

### Ethics Statement

The study was carried out in accordance with the Declaration of Helsinki and the study protocol was approved by the research ethics committee of the Leipzig University (reference number 397/20-ek). Written informed consent was obtained from all GPs. They did not receive an incentive for their participation. Patients agreed on participation by voluntary returning the anonymous questionnaire. No personal data besides age and sex were assessed.

### Patient Questionnaire

The patient questionnaire was self-developed in the DGP of the Leipzig University by an interdisciplinary research team (medical scientists, psychologist, and GPs) in a multi-stage revision process. In addition, an extensive literature research aiming at identifying relevant factors for the progression of CAD and behavioral interactions complemented the questionnaire development. The final version can be found in **Supplementary Material S1**. It is composed of the following topics: (1) demographics (e.g., age, sex, education), (2) information on health behavior and medical history (e.g., other chronic diseases, smoking and drinking habits, physical activity), (3) dealing with risk of infection (e.g., comply with hygiene measures), (4) consultation of medical services during the coronavirus epidemic and comparison with pre-pandemic consultations, (5) symptoms of heart disease since the outbreak of the epidemic (e.g., chest pain, shortness of breath, severity of symptoms), (6) psychological well-being.

Education was assessed by using the CASMIN educational classification (32). Additionally, we assessed state anxiety by using a German version of the Generalized Anxiety Disorder-7 questionnaire [GAD-7, (33)], which has a maximum score of 21. A total score of ≥ 10 indicates the presence of an anxiety symptomatology. Further, we assessed depressive symptoms by using a German version of the Patient Health Questionnaire [PHQ-9, (34)], which has a maximum score of 27. A total score of ≥ 5 indicates the presence of depressive symptoms. In both questionnaires, data were missing at random (GAD-7: in 13.1% of cases; PHQ-9: in 11.1% of cases). In case missing values for GAD-7 or PHQ-9 accounted for <30% of all items (one or two items per questionnaire), individual mean was imputed (GAD-7: *n* = 44; PHQ-9: *n* = 43) (35). Besides the GAD-7 and PHQ-9 scales, missing data of other items in the questionnaire were not imputed and results were presented as percentage of valid cases.

The questionnaire underwent a think-aloud pre-testing (36) aiming at identifying problems or misunderstandings related to each item and was adjusted afterwards. The provisional questionnaire was filled out by five DMP-CAD patients. They were instructed to think aloud while answering each item and report every spontaneous thought. After completing the questionnaire, the patients were shortly interviewed about general issues with the questionnaire (e.g., length, font size, structure, and general comprehensibility). The interviews were tape-recorded. After the think-aloud pre-testing, the provisional questionnaire was adjusted and further developed: nine items were removed due to recurrent misunderstandings (e.g., medical terms) or due to low relevance for the patients. Four items were added (according to patients' suggestions). Twenty-two items were revised and simplified (e.g., changes in terms and wording).

### Measures of CAD Symptom Severity and Risk Stratification

Patients' reports on CAD symptoms and their severity were transformed into a scoring system. The patient questionnaire assessed whether patients suffered from chest pain or dyspnea as characteristic CAD symptoms since the outbreak of the COVID-19 pandemic. To classify patients' symptoms depending on the situation of occurrence, patients were asked whether their symptoms occurred during physical or emotional stress, moderate physical activity, or at rest. The severity of the patients' CAD symptoms was then classified based on the Canadian Cardiovascular Society classification for angina pectoris (CCSA) (37, 38). The CCSA describes four grades of severity depending on the physical activity threshold at which angina symptoms occur. Grade I to IV of the CSSA classification were equivalently matched with CAD symptoms reported in this study (Table 3).

**Table 3:** Assignment of patients to CSSA grades.

**CCSA grade**	**Definition of CCSA grade**	**Assignment based on CAD symptoms reported in this study**
Grade I	No CAD symptoms in everyday life	Patients who did not report CAD symptoms (assuming them to have grade I as they are enrolled in DMP-CAD)
Grade II	CAD symptoms in situations of physical exertion	Patients who reported chest pain and/or dyspnea related to physical or emotional stress
Grade III	CAD symptoms in situations of moderate physical activity	Patients who reported chest pain and/or dyspnea related to moderate physical activity
Grade IV	CAD symptoms at rest or slightest physical activity	Patients who reported chest pain and/or dyspnea at rest

*CAD, coronary artery disease; CCSA, Canadian Cardiovascular Society Angina Classification; DMP, Disease Management Program*.

In addition to the classification of CAD symptom severity, we further carried out a risk stratification. We assessed whether chest pain or dyspnea improved at rest within a maximum of 20 min. Symptoms at rest longer than 20 min are associated with a prolonged cardiac ischemic episode and are therefore described as a risk factor for an unstable angina pectoris (38–40). In this study, patients with a CCSA score between II and IV who reported no improvement of symptoms at rest within 20 min were classified as high risk. Patients with no symptoms or a CCSA score between II and IV who reported an improvement of symptoms at rest within 20 min were classified as low risk.

### Statistical Analyses

All statistical analyses were carried out using IBM SPSS Statistics 25 (Armonk, NY, USA) with a two-sided α-level of 0.05. For descriptive statistics, missing values in single variables were considered by presenting frequencies as % (n/n_valid_). Continuous variables were presented as mean ± standard deviation (SD). Group differences in categorial variables were analyzed using chi-square or Fisher's exact tests, respectively. Estimated effect sizes were reported using Phi (ϕ) or Cramer's V, depending on number of categories.

Changes in quarterly DMP consultations reported by participating GPs were analyzed using repeated-measures analyses of variance (ANOVA) for all time points. Group differences in continuous variables of the patient reports–including age, being worried about COVID-19 (values 1–5), perceived influence on COVID-19 infection risk (values 0–10), feeling threatened by COVID-19 due to risk of infection (values 1–5), feeling threatened by COVID-19 due to age (values 1–5), feeling threatened by COVID-19 due to pre-existing health conditions (values 1–5), anxiety (GAD-7), and depression (PHQ-9)—were analyzed using univariate ANOVAs. Estimated effect sizes were reported using partial eta squared ($\eta_{\text{p}}^{2}$).

## Results

### Sample Characteristics

GP and patient sample characteristics are given in Tables 1, 2. Descriptive values for the total sample as well as for age subgroups are listed. Table 1 contains general characteristics of participating GPs as well as alternative consultation options that were offered to patients during the pandemic. Table 2 contains general characteristics of participating CAD patients and differences between age groups.

### CAD Patients' Use of Health Care During the COVID-19 Pandemic

Since the outbreak of the COVID-19 pandemic, reports of participating GPs indicated a non-significant increase of quarterly DMP-CAD consultations (*F*_(1, 16)_ = 4.021, *p* = 0.062, η^2^ = 0.201) in 2020 (*M* = 87.2, SD = 98.1) compared to 2019 (*M* = 82.1, SD = 89.9; Table 1).

In addition, patients were asked if they generally consulted their GP less frequently compared to pre-pandemic times (item 26), which 9.6% of participating CAD patients affirmed. We further examined characteristics of patients that affirmed this statement compared to patients who did not reduce their general GP consultations since the pandemic. We found that these patients also reported reduced physical activity (χ^2^(4) = 21.335, *p* <0.001, *V* = 0.135), reduced family visits (χ^2^[4] = 13.025, *p* = 0.011, *V* = 0.129), and felt more threatened by COVID-19 due to risk of infection (*F*_(1, 718)_ = 5.058, *p* = 0.025, η^2^ = 0.007) as well as due to their age (*F*_(1, 720)_ = 5.283, *p* = 0.022, η^2^ = 0.007).

Further, 17.9% of CAD patients did not keep their regular DMP appointments during the COVID-19 pandemic (item 28). Among the patients who already had DMP appointments, 1.4% canceled and 5.6% postponed the appointments since March 2020 (item 29). Reasons for postponement or cancellation were fear of infection with COVID-19 (32.8%), restricted mobility (19.7%), and unexpected events (18.0%). Patients who postponed or canceled their appointments exhibited higher anxiety than patients who kept their appointments (*F*_(1, 683)_ = 4.611, *p* = 0.032, η^2^ = 0.007). More information on CAD patients' use of health care can be found in **Supplementary Material S2**.

### Use of Health Care by Symptomatic CAD Patients During the COVID-19 Pandemic

We explored if the fear of getting infected with COVID-19 influenced the decision to consult a doctor in CAD patients with acute symptoms of their heart disease. Patients were asked if they avoided medical care since March 2020 despite symptoms of their heart disease out of concern about the risk of infection (item 37). Importantly, 9.1% of the patients affirmed this statement. In comparison to patients who answered in the negative, they stated lower perceived own influence on COVID-19 infection risk (e.g., through hygiene measures) and exhibited higher anxiety scores (Table 4).

**Table 4:** Use of health care by symptomatic CAD patients during the COVID-19 pandemic.

	**Patients with general CAD symptoms**			**Patients with chest pain longer than 30 min**		
	**Consulted a doctor**	**Did not consult a doctor**	**Statistics**	**Consulted a doctor**	**Did not consult a doctor**	**Statistics**
	***n* = 478**	***n* = 48**		***n* = 54**	***n* = 23**	
Age	73.21 ± 0.4	72.91 ± 0.1	*F*_(1, 512)_ = 0.034	71.99 ±.3	72.51 ± 0.0	*F*_(1, 73)_ = 0.068
			*p* = 0.853			*p* = 0.795
			η^2^ <0.001			η^2^ = 0.001
Sex	34.7%♀	41.3%♀	χ^2^[1] = 0.800	33.3%♀	23.8%♀	χ^2^[1] = 0.626
			*p* = 0.371			*p* = 0.429
			ϕ = 0.041			ϕ = 0.095
Education						
Primary	3.2%	8.5%	χ^2^[2] = 3.249	2.0%	4.3%	χ^2^[2] = 0.718
Secondary	79.1%	76.6%	*p* = 0.177	76.0%	73.9%	*p* = 0.889
Tertiary	17.7%	14.9%	V = 0.082	22.0%	21.7%	V = 0.067
CAD symptoms (CCSA)						
Grade I	53.3%	48.9%	χ^2^[3] = 3.521	13.5%	4.5%	χ^2^[3] = 8.835
Grade II	13.2%	17.0%	*p* = 0.318	7.7%	31.8%	***p*** **=** **0.024**
Grade III	20.0%	12.8%	V = 0.084	34.6%	13.6%	V = 0.362
Grade IV	13.6%	21.3%		44.2%	50.0%	
Proportion of patients with high angina pectoris risk	9.2%	14.9%	χ^2^[1] = 1.565	34.6%	31.8%	χ^2^[1] = 0.054
			*p* = 0.211			*p* = 0.816
			ϕ = −0.056			ϕ = −0.027
Being worried about COVID-19	3.81 ±.1	3.91 ±.4	*F*_(1, 516)_ = 0.747	3.51 ±.2	4.10 ±.8	*F*_(1, 73)_ = 4.043
			*p* = 0.388			***p*** **=** **0.048**
			η^2^ = 0.001			η^2^ = 0.052
Perceived own influence on COVID-19 infection risk	7.12 ±.8	6.13 ±.3	*F*_(1, 514)_ = 4.952	7.52 ±.6	7.42 ± .6	*F*_(1, 73)_ = 0.023
			***p*** **=** **0.027**			*p* = 0.880
			η^2^ = 0.010			η^2^ <0.001
Feeling threatened due to risk of infection	3.81 ±.1	3.61 ±.1	*F*_(1, 519)_ = 0.463	3.51 ±.2	3.81 ±.1	*F*_(1, 74)_ = 0.890
			*p* = 0.497			*p* = 0.348
			η^2^ = 0.001			η^2^ = 0.012
Anxiety (GAD-7)	4.24 ±.2	5.44 ±.1	*F*_(1, 496)_ = 3.912	5.14 ±.5	6.25 ± .5	*F*_(1, 69)_ = 0.775
			***p*** **=** **0.048**			*p* = 0.382
			η^2^ = 0.008			η^2^ = 0.011
Depression (PHQ-9)	4.54 ±.2	5.64 ±.4	*F*_(1, 507)_ = 3.367	5.34 ±.5	6.75 ±.7	*F*_(1, 72)_ = 1.297
			*p* = 0.067			*p* = 0.259
			η^2^ = 0.007			η^2^ = 0.018

*Data are presented as mean, standard deviations, and percentage (n/n_valid_). CAD, coronary artery disease; CCSA, Canadian Cardiovascular Society Angina Classification; GAD-7, Generalized Anxiety Disorder Questionnaire; PHQ-9, Patient Health Questionnaire. The bold p-values highlight a significant result*.

In addition, patients were asked whether they experienced chest pain lasting longer than 30 minutes since March 2020 (item 34), which 10.6% of the patients affirmed. Among these symptomatic patients, 29.9% did not consult a medical practitioner subsequently. It was further investigated how these patients differed in other variables from symptomatic patients who sought medical help. Strikingly, they were more worried about COVID-19 (Table 4).

### Severity of Symptoms of CAD Patients Since the Outbreak of the COVID-19 Pandemic

We further assessed CAD symptoms and potential changes of these symptoms since the outbreak of the pandemic (item 33, 35, 36). In our sample, 21.9% of the CAD patients reported chest pain, 33.3% dyspnea, and 14.2% perspiration or nausea without apparent reason since the outbreak of the COVID-19 pandemic. Of patients with chest pain, 7.5% reported an improvement, 84.2% reported no change, and 8.3% reported a worsening of symptoms since March 2020. Of patients with dyspnea, 4.9% reported an improvement, 85.2% reported no change, and 9.9% reported a worsening of symptoms since March 2020. Of patients with perspiration or nausea without apparent reason, 2.7% reported an improvement, 86.7% reported no change, and 10.7% reported a worsening of symptoms since March 2020.

**Figure 3:**
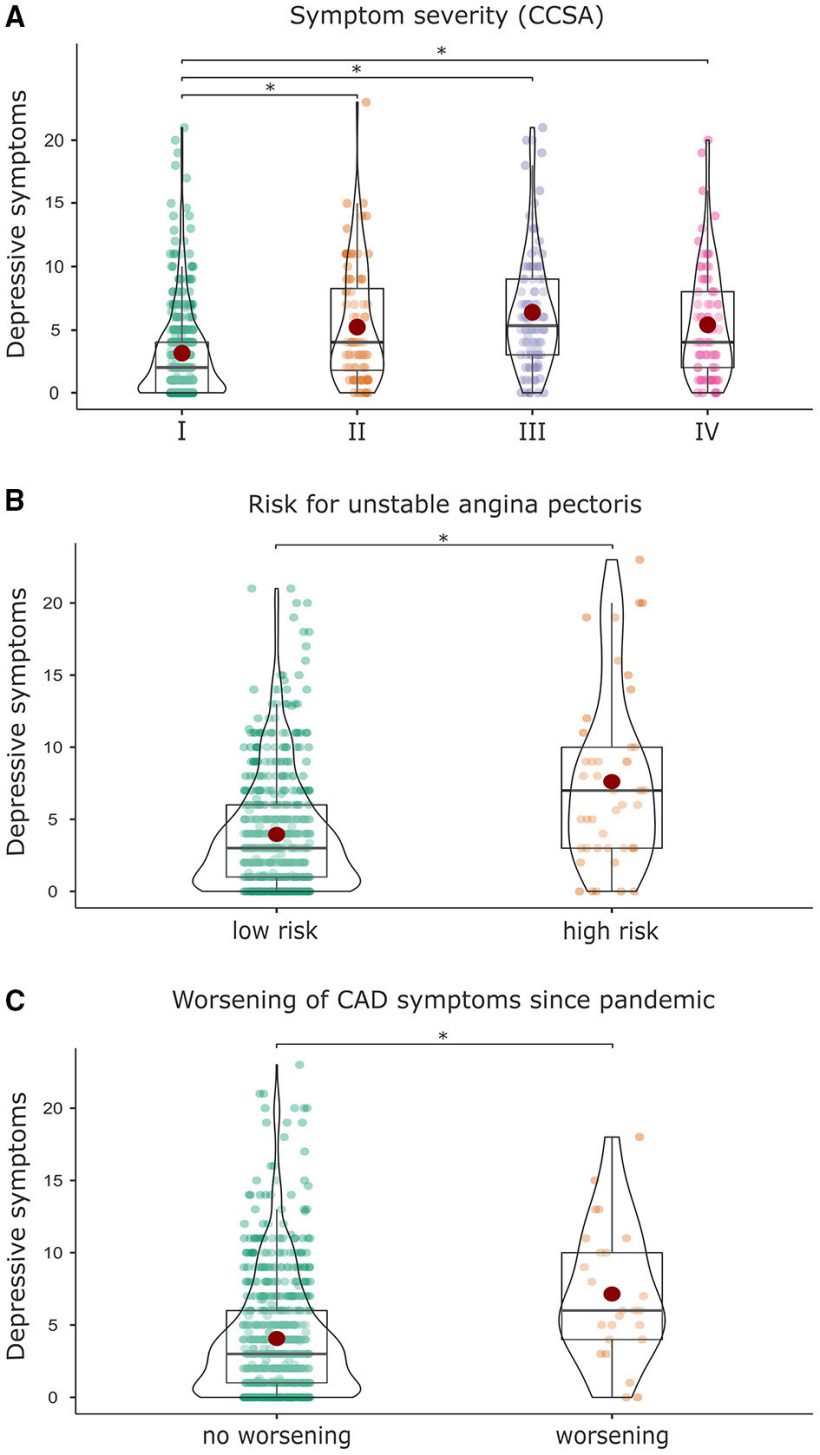
Association of depressive symptoms with **(A)** higher symptom severity measured with the Canadian Cardiovascular Society Angina Classification (CCSA), **(B)** higher risk for an unstable angina pectoris, and **(C)** a worsening of CAD symptoms since the outbreak of the pandemic.

Then, characteristics of patients with at least one worsening CAD symptom since the pandemic were examined: compared to patients with no worsening CAD symptoms, they exhibited more depressive symptoms (*F*_(1, 708)_ = 13.468, *p* <0.001, η^2^ = 0.019; Figure 3C), higher anxiety scores (*F*_(1, 694)_ = 11.107, *p* = 0.001, η^2^ = 0.016), and a higher CCSA grade (χ^2^(3) = 25.052, *p* <0.001, *V* = *0.1*96). Importantly, they were more likely to not consult a medical doctor despite having CAD symptoms out of concern about getting infected with COVID-19 (χ^2^(1) = 5.687, *p* = 0.017, φ = 0.104) and reported leaving their homes less frequently than before the pandemic (χ^2^(1) = 7.695, *p* = 0.006, ϕ = −0.102) compared to patients with no worsening CAD symptoms.

Further, the severity of CAD symptoms according to the CCSA classification grade I-IV and their frequencies in our patient sample can be found in Table 5. Patients with a higher CCSA score also showed a higher risk for an unstable angina pectoris measured by symptoms lasting longer than 20 min at rest (χ^2^(3) = 101.218, *p* <0.001, *V* = 0.359; Table 5).

**Table 5:** Proportion of patients with high and low risk for an unstable angina pectoris in each of the CCSA grades.

**CCSA grade**	**Total number of patients (*n*)**	**Percentage of patients (%)**	**Low risk (%)**	**High risk (%)**
Grade I	410	58.6	100.0%	0.0%
Grade II	88	12.6	88.6%	11.4%
Grade III	120	17.1	80.0%	20.0%
Grade IV	82	11.7	76.8%	23.2%

*Information on symptoms of heart disease were not completely available for 50 patients*.

In addition, patients with a CCSA grade I had significantly lower depressive symptoms (*F*_(3, 662)_ = 23.980, *p* <0.001, η^2^ = 0.098, *M* = 3.1, *SD* = 3.6) compared to patients with a CCSA grade II (*M* = 5.2, *SD* = 4.5), III (*M* = 6.4, *SD* = 4.6), or IV (*M* = 5.3, *SD* = 4.4; Figure 3A). Patients with a CCSA grade I also had significantly lower anxiety (*F*_(3, 648)_ = 15.440, *p* <0.001, η^2^ = 0.067, *M* = 3.0, *SD* = 3.7) compared to patients with a CCSA grade II (*M* = 5.4, *SD* = 4.9), III (*M* = 4.7, *SD* = 4.3), or IV (*M* = 5.4, *SD* = 4.1).

In line with these results, patients with higher risk for angina pectoris scored higher in anxiety (*F*_(1, 650)_ = 28.730, *p* <0.001, η^2^ = 0.042, *M* = 7.0, *SD* = 5.6) and depressive symptoms (*F*_(1, 664)_ = 31.110, *p* <0.001, η^2^ = 0.045, *M* = 7.3, *SD* = 5.9) in comparison to low risk patients (GAD-7: *M* = 3.7, *SD* = 3.9; PHQ-9: *M* = 3.9, *SD* = 3.9; Figure 3B).

### Health Behavior Since the Outbreak of the COVID-19 Pandemic

Since March 2020, the majority of patients reported an unchanged health behavior (items 14–17) with respect to physical activity (82.3%), sports (62.2%), smoking (75.4%), and moderate consumption of alcohol (76.4%). More information on changes in health behavior since March 2020 are given in Table 6. In addition, most participants regarded enhanced standards of hygiene (91.7%), a safety distance of 1.5 m (86.4%), as well as wearing oronasal masks (83.0%) as very important measures to prevent the further spread of the COVID-19 disease (item 19).

**Table 6:** Health behavior of CAD patients.

	**Total**	**Age group**	**Age group**	**Age group**	**Age group**
		**40–59**	**60–79**	**80–99**	**comparison**
*n*	750	83	400	242	
Smoking	9.8%	31.3%	10.3%	1.3%	χ^2^[2] = 56.797
					***p** **<*** **0.001**
					V = 0.296
Smoking since pandemic					
More	4.3%	8.3%	2.6%	0.0%	χ^2^[4] = 4.957
Unchanged	75.4%	83.3%	71.8%	66.7%	*p* = 0.263
Less	20.3%	8.3%	25.6%	33.3%	V = 0.176
Moderate alcohol consumption	59.7%	70.4%	63.9%	50.2%	χ^2^[2] = 15.499
					***p** **<*** **0.001**
					V = 0.148
Moderate consumption of alcohol since pandemic					
More	1.2%	3.4%	1.3%	0.0%	χ^2^[4] = 4.250
Unchanged	76.4%	77.6%	77.1%	74.1%	*p* = 0.326
Less	22.4%	19.0%	21.6%	25.9%	V = 0.076
Member of cardiac exercise group	9.1%	12.7%	10.0%	6.6%	χ^2^[2] = 3.355
					*p* = 0.187
					V = 0.070
Other sports	58.2%	55.1%	67.7%	43.3%	χ^2^[2] = 33.595
					***p** **<*** **0.001**
					V = 0.225
Sports since pandemic					
More	3.9%	10.9%	3.7%	0.3%	χ^2^[4] = 10.402
Unchanged	62.2%	63.0%	64.0%	55.1%	***p*** **=** **0.027**
Less	33.9%	26.1%	32.2%	43.8%	V = 0.125
Physical activities since pandemic					
More	3.8%	3.7%	5.2%	1.7%	χ^2^[4] = 6.009
Unchanged	82.3%	85.2%	81.9%	82.2%	*p* = 0.190
Less	13.9%	11.1%	13.0%	16.1%	V = 0.066
Interested in getting COVID-19 vaccination					
Yes	64.3%	46.3%	63.5%	72.9%	χ^2^[4] = 23.240
Unsure	26.5%	35.0%	28.6%	20.1%	***p** **<*** **0.001**
No	9.1%	18.8%	7.9%	7.0%	V = 0.130
Influenza vaccination					
Yes, annually	65.1%	36.3%	63.8%	76.9%	χ^2^[6] = 49.639
Usually	5.7%	7.5%	5.9%	4.8%	***p** **<*** **0.001**
Sometimes	7.6%	13.8%	9.8%	2.6%	V = 0.189
Never	21.6%	42.5%	20.4%	15.7%	

*Data are presented as mean, standard deviations, and percentage (n/n_valid_). Age was not available for 25 patients. The bold p-values highlight a significant result*.

We further investigated characteristics of CAD patients who reported at least one worsening health behavior since the outbreak of the pandemic (smoking more: *n* = 3, drinking more alcohol: *n* = 5, reduced sports: *n* = 132, reduced physical activity: *n* = 100): compared to patients with unchanged or improved health behavior, they showed more depressive symptoms (*F*_(1, 683)_ = 12.899, *p* < 0.001, η^2^ = 0.019), higher anxiety scores (*F*_(1, 671)_ = 9.368, *p* = 0.002, η^2^ = 0.014), and reported less family visits (χ^2^(2) = 21.663, *p* <0.001, *V* = 0.197). Further, they reported being more worried about COVID-19 (*F*(1, 694) = 6.772, *p* = 0.009, η^2^ = 0.010) and felt more threatened by the risk of a COVID-19 infection (*F*_(1, 710)_ = 6.014, *p* = 0.014, η^2^ = 0.008).

### Mental Well-Being Since the Outbreak of the COVID-19 Pandemic

We also examined mental well-being during the pandemic in CAD patients. In the overall sample, scores in PHQ-9 and GAD-7 were below the thresholds indicating depressive symptoms or anxiety symptomatology. Women showed more depressive symptoms and anxiety compared to men (PHQ-9: *F*_(1, 637)_ = 20.330, *p* <0.001, η^2^ = 0.031; GAD-7: *F*_(1, 626)_ = 30.607, *p* <0.001, η^2^ = 0.047). Additional information on mental well-being can be found in Table 7.

**Table 7:** Mental health of CAD patients.

	**Total**	**Age group 40–59**	**Age group 60–79**	**Age group 80–99**	**Age group comparison**
*n*	750	83	400	242	
Anxiety (GAD-7)	3.9 ± 4.1	4.2 ± 4.3	4.2 ± 4.3	3.4 ± 3.5	*F* = 2.709
					*p* = 0.067
					η^2^ = 0.008
Depression (PHQ-9)	4.2 ± 4.2	3.8 ± 4.0	4.3 ± 4.4	4.3 ± 3.8	*F* = 0.546
					*p* = 0.580
					η^2^ = 0.002
Perceived influence on COVID-19 infection risk	7.0 ± 2.8	6.5 ±2.9	7.2 ±2.7	7.0 ±2.9	*F* = 2.507
					*p* = 0.082
					η^2^ = 0.007
Being worried about COVID-19	3.7 ± 1.1	3.6 ±1.1	3.7 ±1.1	3.8 ±1.1	*F* = 1.317
					*p* = 0.269
					η^2^ = 0.004
Frequency of leaving home between March and May 2020					
Higher frequency	2.2%	3.7%	1.8%	2.1%	χ^2^(4) = 3.881
Same frequency	59.6%	65.4%	57.6%	59.3%	*p* = 0.467
Lower frequency	38.3%	30.9%	40.7%	38.6%	V = 0.050
Emotional stress due to lower frequency of leaving home					
Highly stressed	4.9%	11.9%	4.1%	4.7%	χ^2^(7) = 8.113
Moderately stressed	34.3%	42.9%	32.5%	36.5%	*p* = 0.230
Hardly stressed	37.7%	31.0%	39.9%	35.1%	V = 0.097
Not stressed	23.1%	14.3%	23.5%	23.6%	
Current frequency of leaving home					χ^2^(2) = 4.275
As often as before pandemic	60.7%	70.7%	58.9%	58.7%	*p* = 0.118
Still lower than before pandemic	39.3%	29.3%	41.1%	41.3%	V = 0.078
Social support available in case of emergency					
Strongly agree	64.4%	65.9%	61.4%	69.0%	χ^2^(7) = 8.865
Agree	18.8%	24.4%	19.4%	15.9%	*p* = 0.177
Partly agree	10.8%	4.9%	12.3%	10.8%	V = 0.080
Disagree	6.0%	4.9%	6.8%	4.3%	
Family visits since pandemic: yes	79.0%	82.7%	80.8%	75.7%	χ^2^(2) = 2.955
					*p* = 0.228
					V = 0.063
More than before	0.9%	3.0%	0.7%	0.2%	χ^2^(4) = 3.991
Unchanged	49.7%	52.2%	50.0%	48.8%	*p* = 0.407
Less than before	49.4 %	44.8%	49.3%	50.6%	V = 0.061
Emotional stress due to lack of social activities					
Highly stressed	9.6%	17.3%	9.7%	7.2%	
Moderately stressed	20.8%	22.2%	21.8%	18.6%	χ^2^(11) = 19.422
Partly stressed	24.4%	18.5%	25.6%	23.3%	***p*** **=** **0.035**
Hardly stressed	17.2%	21.0%	18.5%	15.3%	V = 0.167
Not stressed	8.8%	8.6%	7.7%	9.7%	
Not applicable	19.3%	12.3%	16.7%	25.8%	

*Data are presented as mean, standard deviations, and percentage (n/n_valid_). Age was not available for 25 patients. GAD-7, Generalized Anxiety Disorder Questionnaire, PHQ-9, Patient Health Questionnaire. The bold p-values highlight a significant result*.

**Figure 4:**
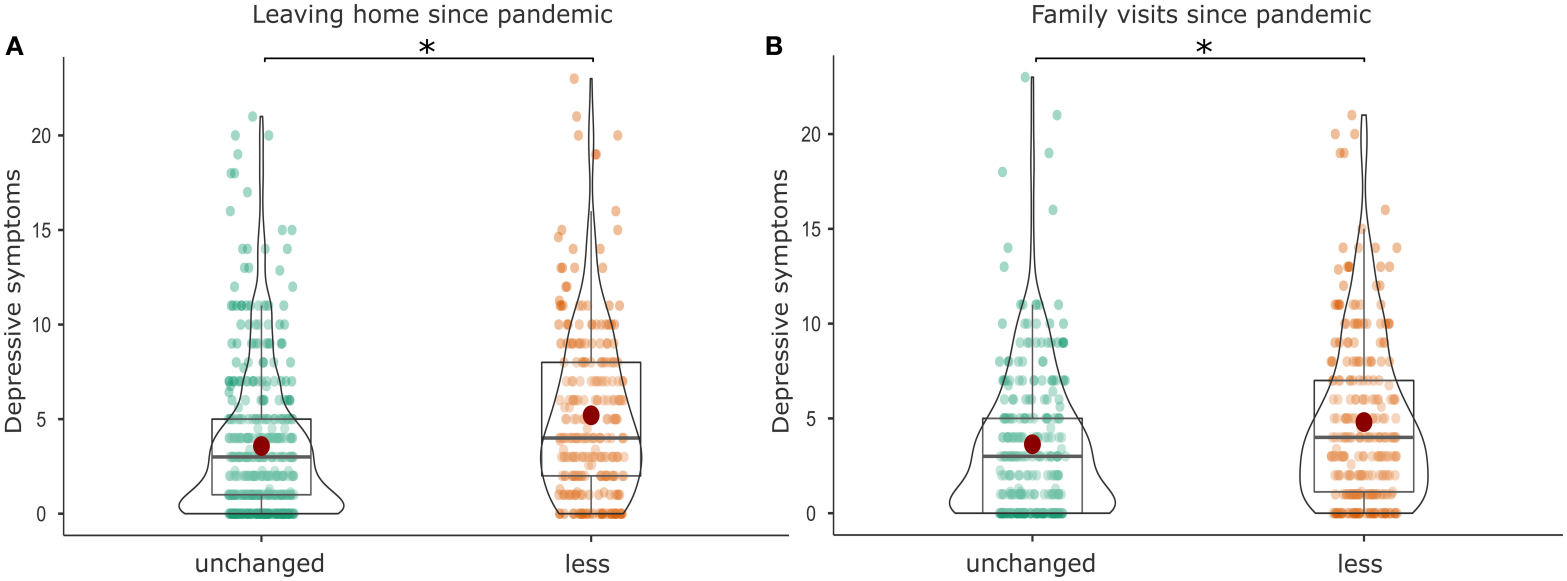
Association of depressive symptoms with **(A)** a reduced frequency of leaving home since the pandemic and **(B)** a reduced frequency of family visits since the pandemic. *Highlights a significant difference between the results.

We further explored how depressive symptoms or anxiety were related to other variables. Patients scoring higher in depression and anxiety were more likely to report less family visits compared to patients with unchanged frequency of family visits (PHQ-9: *F*_(1, 529)_ = 10.883, *p* <0.001, η^2^ = 0.020; Figure 4B; GAD-7: *F*_(1, 523)_ = 14.864, *p* <0.001, η^2^ = 0.028) and further reported a lower frequency of leaving their homes compared to patients who reported leaving their homes as often as before the pandemic (PHQ-9: *F*_(1, 702)_ = 39.481, *p* <0.001, η^2^ = 0.053; Figure 4A; GAD-7: *F*_(1, 689)_ = 38.901, *p* <0.001, η^2^ = 0.053).

**Figure 5:**
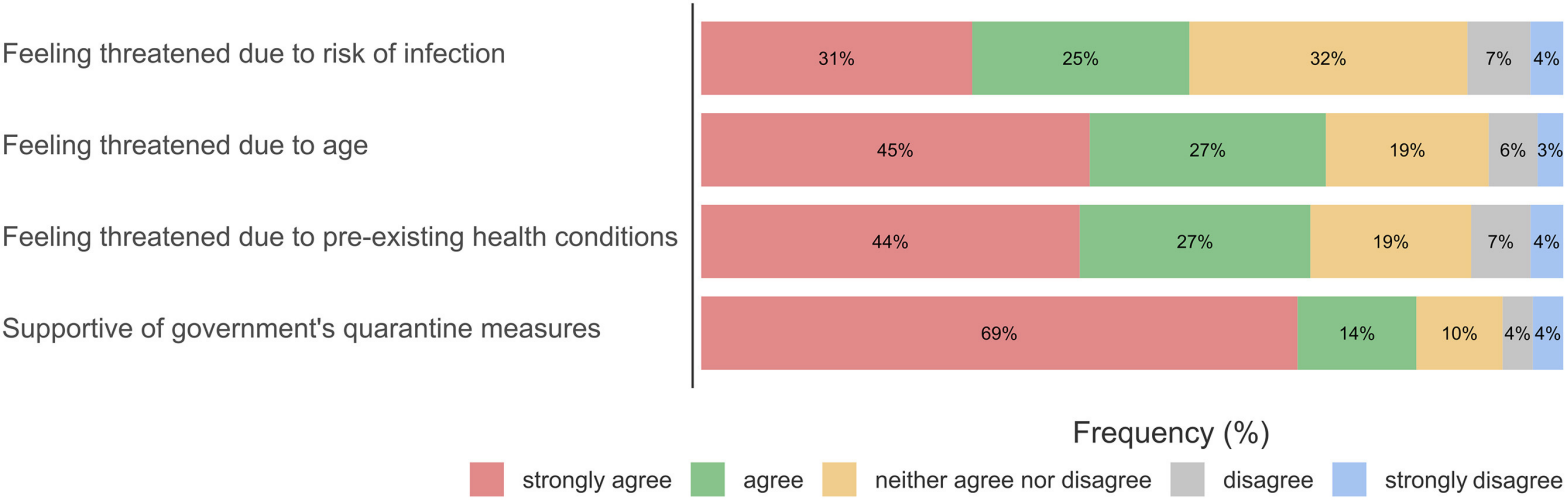
Attitude toward COVID-19 in consideration of individual risks.

In addition, the frequency of family visits was also reduced in CAD patients who felt more threatened by the coronavirus in particular due to their age (*F*_(1, 544)_ = 20.865, *p* <0.001, η^2^ = 0.037). Similarly, the frequency of leaving their homes was reduced in CAD patients who felt more threatened by the coronavirus in particular due to pre-existing health conditions (*F*_(2, 725)_ = 16.439, *p* <0.001, η^2^ = 0.043). Frequencies of perceived threat by the coronavirus can be found in Figure 5.

## Discussion

The present study investigated the effects of the COVID-19 pandemic on health behavior and health outcomes of 750 CAD patients treated within a DMP in Germany. We found that out of concern about getting infected with COVID-19, nearly one tenth of patients did not consult a GP despite having CAD symptoms. These patients reported more often a worsening of their CAD symptoms and exhibited higher state anxiety compared to symptomatic patients who consulted a GP. Notably, one third of the patients with chest pain lasting longer than half an hour did not seek medical help subsequently. These patients were more worried about COVID-19 than patients with chest pain who sought medical help. Our results provide evidence that the COVID-19 pandemic influenced the willingness of some CAD patients to seek medical care and those patients who are more concerned about COVID-19 and exhibited higher anxiety are especially vulnerable.

### CAD Patients' Use of Health Care During the COVID-19 Pandemic

We examined CAD patients' adherence to regular medical consultations since the pandemic and explored potential reasons for non-utilization of the health care system. In contrast to clinical (3, 9) and primary care data (41–43), accounting data of participating GPs showed no reduced frequency of quarterly DMP consultations. Additionally, despite the possibility to suspend or cancel DMP interventions since March 2020 (22), we found no evidence that GP practices referred to this option, indicating an ensured provision of medical care for DMP-CAD patients during the pandemic in Central Germany.

The CAD patients' data show that every tenth participant reported to visit his/her GP generally less often than before the pandemic. In contrast, across Europe, four times as many patients with chronic diseases reported visiting their doctor less frequently (44), indicating a comparable mild effect of the pandemic on CAD patients' health care in our sample from Central Germany. There might be two potential reasons for this effect: on the one hand, the high compliance of CAD patients during the pandemic might be attributable to the DMP enrollment, which has previously been found to improve CAD patients' adherence to medication, frequency of medical care, and disease-related knowledge and awareness (20, 21, 45, 46). On the other hand, Central Germany was less affected by the pandemic compared to other European countries, which potentially caused less changes in patients' health behavior (47).

Regarding DMP consultations, one in six CAD patients in our study reported not going to regular DMP consultations any longer since the outbreak of the COVID-19 pandemic, whereas only a minor group of patients who already had a scheduled appointment decided to cancel or postpone it. Specifically, the fear of infection with COVID-19 accounted for one third of postponed or canceled appointments, which has also been observed in other medical sectors (48, 49). This patient group may have tried to cope with the perceived risk due to pre-existing health conditions by a general avoidance behavior toward situations with potentially elevated infection risk—such as doctor's offices –, which has also been reported in other studies (50, 51).

### Use of Health Care by Symptomatic CAD Patients During the COVID-19 Pandemic

In addition to preventive medical consultations, we investigated if patients with clear signs of their CAD sought medical care since the outbreak of the pandemic. Almost one in ten patients affirmed that they avoided medical care since March 2020 despite symptoms of their heart disease out of concern about the infection risk. Even more alarming, almost every third patient who reported chest pain longer than half an hour did not consult a medical practitioner subsequently. In comparison to participants who sought medical help, these patients were generally more worried about COVID-19, indicating that the pandemic might have encouraged some patients with severe CAD symptoms to not seek medical help.

Although most patients know that symptoms of their heart disease, especially chest pain, could develop into a heart attack (52), being worried about a potential infection with COVID-19 might still outweigh the risks of not consulting a medical specialist. Especially patients with a perceived high risk for severe COVID-19, e.g., due to underlying medical conditions, have been found to more often avoid medical care (48, 53). Clinical data reported similar findings: patients with AMI stated COVID-19-related concerns as reasons for presenting delayed at hospital (12, 18).

### Severity of Symptoms of CAD Patients Since the Outbreak of the COVID-19 Pandemic

We further explored patients' general CAD symptom severity and whether they experienced changes in severity since the pandemic started. In general, every tenth patient exhibited severe CAD symptoms classified as CCSA grade IV. In addition, patients with a higher CCSA grade reported more often symptoms that might be an indicator for an unstable angina pectoris and that might require treatment, such as chest pain longer than 30 min. It has been shown that patients with CCSA grade III or IV with an unstable course have a mortality rate of 8.1% within 1 year (23). Similarly to a recent study (54), our results indicate that there might be a considerable prevalence of primary care CAD patients who are not optimally controlled. Although the DMP-CAD enrollment significantly reduces patients' mortality and improves medication adherence and adjustment (19–21), our results suggest potential for improvements and highlight the need for better communication strategies between patient and physician.

The majority of patients stated unchanged symptoms since March 2020. However, every tenth patient reported a worsening of their CAD symptoms. A key finding of our study was that patients with at least one worsening CAD symptom since March 2020—compared to patients without worsening of symptoms—consulted a doctor less often despite having CAD symptoms to avoid the risk of infection with COVID-19. Our findings are in line with clinical data reporting increased severe AMI cases since the outbreak of the pandemic, suggesting a delayed treatment-seeking in patients (6, 8, 9, 55). In other patient groups, worse health outcomes due to delayed medical care utilization have also been observed (56–58). Avoiding medical care even when necessary has been associated with higher psychological distress and less help with uncertainty (57, 59). Since the pandemic began, symptomatic patients might feel an uncertainty about their individual vulnerability to COVID-19 and weigh the risk of a potential infection against the consequences of not seeking medical help (60). Our results suggest that the trade-off might increase the risk of worsening symptoms and adversely affect patients' long-term health outcomes.

### Mental Well-Being Since the Outbreak of the COVID-19 Pandemic

We also examined patients' psychological distress, e.g., depressive symptoms or anxiety, and their influence on regular GP consultations, treatment-seeking in case of CAD symptoms, or symptom severity. Our data showed that CAD patients with more depressive symptoms and anxiety were more likely to be female, exhibited a higher CCSA grade, a higher risk for an unstable angina, and reported more often a worsening of CAD symptoms since the outbreak of the pandemic. Previous research had established the association between depressive symptoms and severity of CAD, especially in women (29), indicating that the pathophysiological mechanisms of both diseases reinforce each other (27, 30, 61). In line with these findings, CAD patients with anxiety have a higher risk for AMI or mortality within a 3.4 year follow-up period (26, 28). As negative life events were associated with increased depressive symptoms in patients with CAD (62), the COVID-19 pandemic might have added to the mental burden of CAD patients and, therefore, contributed to a worsening of symptoms. Indeed, in the general population, an increase in depressive symptoms and anxiety has been observed since March 2020 compared to pre-pandemic measurements (63, 64). CAD patients might benefit from treatment for depression, as previous research demonstrated that reductions in depressive symptoms mitigate CAD symptoms (61).

Further, we found that CAD patients with more anxiety were more likely to avoid medical care. Previously, patients with symptoms of depression or anxiety have been found to be twice as likely to avoid medical care since the COVID-19 pandemic despite being necessary (65). Avoidance of or inconsistent utilization of health care have also been reported independently of the COVID-19 pandemic in patients with psychological distress and anxiety (66, 67). Therefore, patients with signs of worries and anxiety might benefit from greater attention of the treating physician.

We further found that a decrease in family visits and a lower frequency of leaving homes since the pandemic were associated with decreased GP consultations, worsening of CAD symptoms, more anxiety and depressive symptoms, as well as a higher individual risk perception. It might be possible that reduced family visits and/or staying at home increased loneliness (64), which has been found recently to be associated with an increased likelihood of canceling medical appointments (50). Further, mental health was more impaired in individuals who reduced their social contacts and who belonged officially to a COVID-19 risk group (62, 64). Our results indicate that social contacts might mitigate the negative impact of the pandemic on mental and physical health.

### Health Behavior Since the Outbreak of the COVID-19 Pandemic

Further, CAD patients' health behavior and potential changes since the pandemic were examined. In our study, the majority of patients did not change their health behavior (physical activity, sports, drinking alcohol, or smoking) since the pandemic, which is in line with other studies (68). Importantly, worsening of health behavior was not associated with a worsening of CAD symptoms, indicating that physical health in CAD patients was more influenced by psychosocial factors than by health behavior during the pandemic.

### Limitations

This study has limitations. First, data were based on patients' self-reports, e.g., to ensure confidentiality and anonymity, symptoms and symptom severity were not compared with the assessment of the treating GP or patients' record. However, previous studies found that perceived symptoms of heart disease correlated with the functional status measured with the NYHA classification (69), indicating good reliability. Future studies should compare patients' reports with clinical data to get more complex information on their clinical status. Second, due to a cross-sectional study design, we do not have data on patients' status before the pandemic and can only depict subjective perception of changes since the pandemic. In addition, patients did not fill in the questionnaire at the same timepoint, instead ranging from October 2020 to February 2021. Temporary differences of lockdown regulations, case incidences of COVID-19, and subjective experiences with the progression of the pandemic may have influenced patients' reports over time. Third, the study sample was collected in Central Germany and differences might exist between federal states in Germany as well as between European countries due to varying case incidences, lockdown and preventive measures. Further, we did not include a control group, such as CAD patients who are not enrolled in a DMP. Differences in health behavior might exist between these groups. Lastly, as the survey could only assess data on living participants, we do not have information on the influence of avoidance of treatment during the pandemic on CAD patients' mortality.

### Implications

Our research contributes to a better understanding of the impact of the COVID-19 pandemic on treatment-seeking, symptom severity, and health behavior in patients with chronic heart diseases enrolled in the DMP-CAD. The results indicate that GPs should actively advise especially anxious and more concerned or depressed patients to take symptoms of their heart disease seriously and to seek medical help despite a potential infection risk. This patient group might also benefit from low-risk treatment options, such as separate office hours or telemedicine. Medical practices should additionally evaluate if their infection protection is in accordance with current recommendations. In addition, pre-scheduled DMP appointments might contribute to a better appointment compliance. As education could reduce the delay of treatment-seeking in CAD patients (70), we recommend including special information on health behavior and compliance with the treatment during the COVID-19 pandemic in the DMP training courses for patients. Further, the implementation of national campaigns to raise awareness for the potential adverse effects of delayed treatment-seeking for acute conditions have been found to be beneficial (17). Independently of the pandemic, our results indicate that the DMP-CAD has room for improvements in terms of symptom identification and treatment. A potential communication barrier between patients and physicians regarding the severity of CAD symptoms should be further evaluated in future studies.

### Conclusion

We confirm that the majority of DMP-CAD patients reported no changes in CAD symptoms and received sufficient medical care during the COVID-19 pandemic in Central Germany. We found evidence that a minor group of patients neglected medical care–despite it being necessary due to worsening of symptoms–since March 2020 and that their decision might have been influenced by the perceived infection risk with COVID-19. This group was also characterized by more state anxiety and depressive symptoms, indicating that reduced psychological well-being might have aggravated COVID-19-related concerns and, thus, contributed to adverse treatment-seeking decisions and health outcomes.

## Data Availability Statement

The raw data supporting the conclusions of this article will be made available by the authors, without undue reservation.

## Ethics Statement

The studies involving human participants were reviewed and approved by Medical research Ethics Committee of the Leipzig University. Written informed consent for participation was not required for this study in accordance with the national legislation and the institutional requirements.

## Author Contributions

AS, MB, SL, SGRH, and TD designed the study. AS and NM prepared the study, collected and analyzed the data, and wrote the manuscript. SM supported the development of the questionnaire. MB, TD, SL, SM, and SGRH reviewed and edited the manuscript. All authors contributed to the article and approved the submitted version.

## Conflict of Interest

The authors declare that the research was conducted in the absence of any commercial or financial relationships that could be construed as a potential conflict of interest.

## Publisher's Note

All claims expressed in this article are solely those of the authors and do not necessarily represent those of their affiliated organizations, or those of the publisher, the editors and the reviewers. Any product that may be evaluated in this article, or claim that may be made by its manufacturer, is not guaranteed or endorsed by the publisher.
