# Supplementary material for: The Impact of the COVID-19 Pandemic on Avoidance of Health Care, Symptom Severity, and Mental Well-Being in Patients With Coronary Artery Disease
Source: Front Med (Lausanne). 2021 Dec 15;8:760265. doi: 10.3389/fmed.2021.760265 (PMC8714893; doi:10.3389/fmed.2021.760265)
Supplement: Supplementary file 1 [file Data_Sheet_1.docx]

**Supplements**

**Supplementary material S1.** Patient questionnaire translated into English.

| Personal information | | | | | | | | |
| --- | --- | --- | --- | --- | --- | --- | --- | --- |
| 1. 1. Age __ __ years | | | | | | | | |
| 2. Sex | □ female | | □ male | | | | | □ diverse |
| 3. What is your highest education? | □ no degree | | | | □ lower secondary education (grade 8 - 9) | | | |
|  | □ vocational qualification | | | | □ upper secondary education (grade 10) | | | |
|  | □ Abitur/University entrance qualification | | | | | | | |
| 4. What is your highest professional qualification? | □ no professional qualification | | | | | □ semi-skilled training | | |
|  | □ vocational training | | | | | □ apprenticeship | | |
|  | □ specialized technical training | | | | | □ university degree | | |
| 5. What is your current occupation? | □ unemployed | | | □ employed | | | □ retired | |
| 6. If you are still working, are you currently working from home? | □ yes, entirely | | | | □ yes, 3-4 times per week | | | |
|  | □ yes, 1-2 times per week | | | | □ no | | | |
| 7. What is your current housing situation? (*multiple responses possible*) | □ living alone | | | | □ living with spouse/partner | | | |
|  | □ living with child(ren) | | | | □ other flat-sharing community | | | |
| 8. How would you describe your place of residence? | | □ city | | | □ small town | | | □ rural |
| 9. Are you currently living in a district with high risk of COVID-19 infection (was there any official warning)? | | □ yes | | | □ no | | | □ don‘t know |

| Health information | | | |
| --- | --- | --- | --- |
| 10. Do you have a care level? | □ yes | □ no | |
| 11. Do you get any other regular medical care because of another chronic disease? (*multiple responses possible*) | □ no | □ asthma | |
|  | □ COPD | □ type 1 diabetes mellitus | |
|  | □ breast cancer | □ type 2 diabetes mellitus | |
| 12. Are you getting a vaccination against influenza? | □ yes, annually | □ yes, usually | |
|  | □ yes, occasionally | □ no | |
| 13. Would you get a vaccination against COVID-19 if there was a vaccine? | | | |
| □ yes | □ no | □ don’t know | |
| 14. Are you smoking? | □ yes | □ no | |
| **If so,** since the outbreak of the COVID-19 pandemic: | | | |
| □ more often | □ unchanged | □ less often | |
| 15. Are you (occasionally) drinking alcohol? | □ yes | □ no | |
| **If so**, since the outbreak of the COVID-19 pandemic: | | | |
| □ more often | □ unchanged | □ less often | |
| 16. Are you participating in a heart training group? | | □ yes | □ no |
| Did you engage in physical activities (e.g., hiking, biking, etc.) before the COVID-19 pandemic? | | □ yes | |
|  |  | □ no | |
| **If so**, since the outbreak of the COVID-19 pandemic: | | | |
| □ more often | □ unchanged | □ less often | |
| 17. Do you take care of daily physical activities (e.g., climbing stairs, walks) since the onset of the COVID-19 pandemic? | | | |
| □ more than usual | □ unchanged | □ less than usual | |

| Dealing with the risk of a COVID-19 infection | | | | | | | | | | | | | | | | | | | |
| --- | --- | --- | --- | --- | --- | --- | --- | --- | --- | --- | --- | --- | --- | --- | --- | --- | --- | --- | --- |
| 18. Have you already been tested for possible COVID-19 infection with a nasopharyngeal swab? | | | | | | | | | | | | | | | □ yes | | | | |
|  |  |  |  |  |  |  |  |  |  |  |  |  |  |  | □ no | | | | |
| **If so**, where was the swab taken? | | | | | | | | □ fever outpatient department | | | | | | | □ general practitioner | | | | |
|  |  |  |  |  |  |  |  | □ hospital | | | | | | | □ elsewhere | | | | |
| **If so**, was the COVID-19 swab sample positive? | | | | | | | | □ yes | | | | | | | □ no | | | | |
| **19. Since the outbreak of the pandemic, how important do you consider...** | | | | | | | | | | **Very important** | | | | Partly important | | | | Not important | |
| **…** maintaining enhanced hygiene measures (e.g., frequent hand washing with soap, coughing and sneezing with mouth covered)? | | | | | | | | | | □ | | | | □ | | | | □ | |
| **…** keeping a safety distance of 1.5 m from other people? | | | | | | | | | | □ | | | | □ | | | | □ | |
| **…** wearing a face mask in public (especially indoors)? | | | | | | | | | | □ | | | | □ | | | | □ | |
| **20. Will the risk of COVID-19 infection change in the near future in your opinion?** | | | | | | | | □ the risk will decrease | | | | | | | | | | | |
|  |  |  |  |  |  |  |  | □ the risk will increase | | | | | | | | | | | |
|  |  |  |  |  |  |  |  | □ the risk will remain unchanged | | | | | | | | | | | |
| 21. Did you have family visits (e.g., from children or grandchildren) since the outbreak of the pandemic? | | | | | | | | | | | | | | □ yes | | | | | |
|  |  |  |  |  |  |  |  |  |  |  |  |  |  | □ no | | | | | |
| **If so**, since the outbreak of the COVID-19 pandemic: | | | | | | | | | | | | | | | | | | | |
| □ more often | | | | | | □ unchanged | | | | | | □ less often | | | | | | | |
| 22. Do you travel by public transport (e.g., bus, train)? | | | | | | | | | | | | | | □ yes | | | | | |
|  |  |  |  |  |  |  |  |  |  |  |  |  |  | □ no | | | | | |
| **If so**, since the outbreak of the COVID-19 pandemic: | | | | | | | | | | | | | | | | | | | |
| □ more often | | | | | | □ unchanged | | | | | | □ less often | | | | | | | |
| 23. Please tick the statement which matches your opinion | | | | | | | | | | | | | | | | | | | |
| **Totally disagree** | | | **Disagree** | | | **Neither agree nor disagree** | | | | | **Agree** | | | | | **Totally**  **agree** | | | |
| The risk of getting seriously ill with COVID-19 myself worries me. | | | | | | | | | | | | | | | | | | | |
| □ | | | □ | | | □ | | | | | □ | | | | | □ | | | |
| I am particularly threatened by COVID-19 because of my age. | | | | | | | | | | | | | | | | | | | |
| □ | | | □ | | | □ | | | | | □ | | | | | □ | | | |
| I am particularly threatened by COVID-19 because of my pre-existing health-conditions. | | | | | | | | | | | | | | | | | | | |
| □ | | | □ | | | □ | | | | | □ | | | | | □ | | | |
| I absolutely support the government’s measures to prevent the further spread of COVID-19. | | | | | | | | | | | | | | | | | | | |
| □ | | | □ | | | □ | | | | | □ | | | | | □ | | | |
| 24. To what extent do you think you can influence the risk of COVID-19 infection yourself (e.g., by complying with official recommendations)?  **0= no influence 10= total influence** | | | | | | | | | | | | | | | | | | | |
| □  0 | □  1 | □  2 | | □  3 | □  4 | | □  5 | | □  6 | | | □  7 | □  8 | | | | □  9 | | □  10 |
| 25. Since March 2020, have you received informational letters from your health insurance company with specific recommendations for behavior during the pandemic (e.g., doctor’s visit, etc.)? | | | | | | | | | | | | | | | | | □ yes | | |
|  |  |  |  |  |  |  |  |  |  |  |  |  |  |  |  |  | □ no | | |
| **If so**, which recommendations? | | | | | | | | | | | | | | | | | | | |

| Use of the health care system | | | | | | | | | |
| --- | --- | --- | --- | --- | --- | --- | --- | --- | --- |
| **26. How often have you consulted your general practitioner since the outbreak of the COVID-19 pandemic compared to pre-pandemic times?** | | | | | | | □ more often | | |
|  |  |  |  |  |  |  | □ unchanged | | |
|  |  |  |  |  |  |  | □ less often | | |
| 27. How often did you have regular consultations for your heart disease before the COVID-19 pandemic? | | | | | | | □ quarterly | | |
|  |  |  |  |  |  |  | □ bianually | | |
| 28. Have you attended the regular appointments for your heart disease after the onset of the COVID-19 pandemic (March 2020)? | | | | | | | □ yes, quarterly | | |
|  |  |  |  |  |  |  | □ yes, biannually | | |
|  |  |  |  |  |  |  | □ no | | |
| 29. Have you cancelled or postponed already scheduled appointments for your heart disease because of the COVID-19 pandemic? | | | | | | | □ yes, cancelled | | |
|  |  |  |  |  |  |  | □ yes, postponed | | |
|  |  |  |  |  |  |  | □ no | | |
| **If so**, why have you cancelled or postponed appointments? | | | | | | | | | |
| □ out of fear of getting infected in the practice | | | □ something else came up | | | | | | |
| □ restricted mobility | | | □ other reason: _______________ | | | | | | |
| 30. Did your practice cancel appointments since March 2020? | | | | □ yes | | | | □ no | |
| 31. Have any planned medical treatments been cancelled by a hospital or practice because of the COVID-19 pandemic? | | | | | | | | | |
| □ surgery | | □ rehabilitation | | | | □ health cure/resorts | | | |
| □ physiotherapy | | □ others: _____________________ | | | | □ no treatments scheduled | | | |
| 32. Would you like to have the option of low-risk treatments specifically for chronically ill patients because of the COVID-19 pandemic? | | | | | | | | | □ yes |
|  |  |  |  |  |  |  |  |  | □ no |
| **If so**, which would you prefer? | □ separate office hours | | | | □ telephone consultation | | | | |
|  | □ video consultation | | | | □ health applications | | | | |

| Symptoms of the heart disease | | | |
| --- | --- | --- | --- |
| 33. Have you suffered from **pain or discomfort** in your **chest** (maybe also with radiation to the neck, abdomen, or arms) since March 2020? | | | □ yes |
|  |  |  | □ no |
| **If so, did the symptoms occur under strain (physical or emotional)?** | | | □ yes |
|  |  |  | □ no, at rest |
| **If so, did the symptoms occur under light physical activity, e.g., walking at a steady pace?** | | | □ yes |
|  |  |  | □ no |
| **If so, did the symptoms improve on their own after 20 min at rest at the latest?** | | | □ yes |
|  |  |  | □ no |
| **If** **so,** did the symptoms change since the outbreak of the pandemic? | | | |
| □ improved | □ unchanged | □ aggravated | □ don’t know |
| 34. Have you suffered from chest pain longer than half an hour since March 2020? | | | **□** yes |
|  |  |  | □ no |
| **If so,** did you consult a doctor? | | | □ yes |
|  |  |  | □ no |
| 35. Have you suffered from **shortness of breath** since March 2020? | | | □ yes |
|  |  |  | □ no |
| **If so, did the symptoms occur under strain (physical or emotional)?** | | | □ yes |
|  |  |  | □ no, at rest |
| **If** **so, did the symptoms occur under light physical activity, e.g., walking at a steady pace?** | | | □ yes |
|  |  |  | □ no |
| **If** **so, did the symptoms improve on their own after 20 min at rest at the latest?** | | | □ yes |
|  |  |  | □ no |
| **If** **so,** did the symptoms change since the outbreak of the pandemic? | | | |
| □ improved | □ unchanged | □ aggravated | □ don’t know |
| 36. Have you suffered from **sweating** or **nausea** **without** **apparent reason** since March 2020? | | | □ yes |
|  |  |  | □ no |
| **If** **so, did the symptoms occur under strain (physical or emotional)?** | | | □ yes |
|  |  |  | □ no, at rest |
| **If** **so, did the symptoms occur under light physical activity, e.g., walking at a steady pace?** | | | □ yes |
|  |  |  | □ no |
| **If** **so, did the symptoms improve on their own after 20 min at rest at the latest?** | | | □ yes |
|  |  |  | □ no |
| **If** **so**, did the symptoms change since the outbreak of the pandemic? | | | |
| □ improved | □ unchanged | □ aggravated | □ don’t know |
| 37. **If there were symptoms:**  **Did you avoid medical care since March 2020 despite symptoms of your heart disease out of concern about the risk of infection?** | | | □ yes |
|  |  |  | □ no |
| 38. Are you currently able to keep all necessary medical appointments? | | | □ yes |
|  |  |  | □ no |
| **In case of no**, why? | | | |

| Emotional wellbeing | | | |
| --- | --- | --- | --- |
| 39. I am worried about the COVID-19 virus in general. | □ totally disagree | | □ disagree |
|  | □ neither agree nor disagree | | □ agree |
|  | □ totally agree | | |
| 40. From March to May 2020, I have left my home.... | □ more often than before the pandemic. | | |
|  | □ as often as before the pandemic. | | |
|  | □ less often than before the pandemic. | | |
| 41. If you left your home less frequently, how stressful did you perceive this situation? | □ not stressful at all | □ hardly stressful | |
|  | □ quite stressful | □ extremely stressful | |
| 42. How often do you currently leave your home? | □ as often as before the pandemic | | |
|  | □ still less often than before the pandemic | | |
| 43. In case of emergency, I can ask family or friends for help without hesitation. | □ incorrect | □ partly correct | |
|  | □ quite correct | □ absolutely correct | |
| 44. How stressful did you perceive the lack of social events (e.g., family gatherings, concerts, sports) due to the COVID-19 pandemic? | □ the situation did not exist | □ not stressful at all | |
|  | □ hardly stressful | □ partly stressful | |
|  | □ quite stressful | □ extremely stressful | |

| Over the last 2 weeks, how often have you been bothered by the following problems? | | | |
| --- | --- | --- | --- |
| **Not at all** | **Several days** | **More than half the days** | **Nearly every day** |
| Feeling nervous, anxious, or on edge | | | |
| □ | □ | □ | □ |
| Not being able to stop or control worrying | | | |
| □ | □ | □ | □ |
| Worrying too much about different things | | | |
| □ | □ | □ | □ |
| Trouble relaxing | | | |
| □ | □ | □ | □ |
| Being so restless that it is hard to sit still | | | |
| □ | □ | □ | □ |
| Becoming easily annoyed or irritable | | | |
| □ | □ | □ | □ |
| Feeling afraid as if something awful might happen | | | |
| □ | □ | □ | □ |
| Little interest or pleasure in doing things | | | |
| □ | □ | □ | □ |
| Feeling down, depressed, or hopeless | | | |
| □ | □ | □ | □ |
| Trouble falling or staying asleep, or sleeping too much | | | |
| □ | □ | □ | □ |
| Feeling tired or having little energy | | | |
| □ | □ | □ | □ |
| Poor appetite or overeating | | | |
| □ | □ | □ | □ |
| Feeling bad about yourself- or that you are a failure or have let yourself or your family down | | | |
| □ | □ | □ | □ |
| Trouble concentrating on things, such as reading the newspaper or watching TV | | | |
| □ | □ | □ | □ |
| Thoughts that you would be better off dead or hurting yourself in some way | | | |
| □ | □ | □ | □ |
| Moving or speaking so slowly that other people could have noticed or being so fidgety or restless that you have been moving around a lot more than usual | | | |
| □ | □ | □ | □ |

**Supplementary material S2.** CAD patients’ use of health care during the COVID-19 pandemic (n = 750).

| Frequency of general GP appointments since pandemic  More often  Unchanged  Less often | 1.6%  88.8%  9.6% |
| --- | --- |
| Frequency of DMP-CAD appointments before pandemic  Quarterly  Biannually | 56.1%  43.9% |
| Frequency of DMP-CAD appointments since pandemic  Quarterly  Biannually  No longer kept | 48.5%  33.7%  17.9% |
| Patients who already had DMP-CAD appointments since March 2020  Cancelled  Postponed  Kept | 1.4%  5.6%  93.1% |
| Reason for cancelling or postponing DMP-CAD appointments  Fear of infection with COVID-19  Unexpected events  Restricted mobility  Other reason | 32.8%  18.0%  19.7%  29.5% |
| CAD appointment cancelled by practice | 3.3% |
| Other medical treatments cancelled due to pandemic  Nothing cancelled  Surgery  Rehabilitation  Health cure/resorts  Physiotherapy  Others  No treatments scheduled | 15.2%  2.3%  1.6%  0.8%  3.2%  2.5%  74.4% |
| Request for special treatment for patients with chronic disease  Yes  Separate office hours  Telephone consultation  Video consultation  Health applications | 38.0% (268)  72.8% (225/268)  17.8% (55/268)  5.5% (17/268)  3.9% (12/268) |
